# Impact of Probiotics on Atopic Dermatitis in Pediatric Patients: A Systematic Review and Meta-Analysis

**DOI:** 10.3390/medicina61122090

**Published:** 2025-11-24

**Authors:** Ritu Gaikwad, Soham Kondle, Sean Chang, Chris Barnes, Rohan Kubba, Christopher Lane, Snigdha Uppu, Eldo Frezza

**Affiliations:** Department of Clinical Medicine, California Northstate University College of Medicine, Elk Grove, CA 95757, USA; ritu.gaikwad10779@cnsu.edu (R.G.); soham.kondle6396@cnsu.edu (S.K.); sean.chang10272@cnsu.edu (S.C.); chris.barnes12002@cnsu.edu (C.B.); rohan.kubba12079@cnsu.edu (R.K.); christopher.lane8569@cnsu.edu (C.L.); snigdha.uppu11551@cnsu.edu (S.U.)

**Keywords:** probiotics, atopic dermatitis, pediatric, allergy

## Abstract

*Background and Objectives*: Atopic dermatitis (AD) is a chronic inflammatory skin disorder primarily affecting children, driven by genetic, immunologic, and environmental factors. Emerging evidence links gut microbiota alterations to immune modulation and AD severity. Probiotics, live microorganisms providing health benefits when consumed in adequate amounts, have been proposed as a potential adjunctive therapy. This review evaluates the efficacy of various probiotic treatments in reducing SCORAD indices and symptoms in children with AD, and its effects on immunologic markers such as IgE. *Materials and Methods*: Through a systematic literature review of multiple electronic databases through 9 October 2024, we identified randomized controlled trials (RCTs) in pediatric patients with an established diagnosis of atopic dermatitis. Our search strategy was as follows: “((atopy) OR (dermatitis) OR (hypersensitivity)) AND pediatric AND probiotic” yielding 25 total studies. Patients were treated with either a probiotic regimen or placebo and assessed for levels of IgE and SCORAD indices. *Results*: Of 25 studies extracted, 14 RCTs evaluated the effects of probiotics on atopic dermatitis using SCORAD scores. Eleven showed significant reductions in SCORAD indices. Pooled analysis using a random-effects model (Hedges’ *g* ≈ 0.65, *p* < 0.05) indicated a moderate to large improvement in AD severity with probiotic therapy. However, heterogeneity in probiotic strains, intervention duration, and limited sample sizes are limitations that warrant further investigation. Secondary analysis of IgE changes showed a non-significant effect (*g* ≈ 0.15, *p* = 0.13), possibly due to short study durations (mean 12 weeks). *Conclusions*: Probiotics demonstrate a moderate to large clinical impact in reducing SCORAD indices among children with atopic dermatitis. These findings highlight their potential as a future adjunctive, non-pharmaceutical therapy for the roughly 9.6 million pediatric patients affected in the United States. Further studies are needed to clarify strain-specific effects and patient factors influencing response.

## 1. Introduction

Atopic dermatitis (AD) is a chronic inflammatory skin condition characterized by intense pruritus, erythema, and xerosis that often begin in early childhood. It is one of the most common dermatologic conditions worldwide and imposes significant psychosocial and economic burdens [1,2,3]. The pathogenesis of AD is multifactorial, including immune dysregulation, skin barrier dysfunction, genetic predisposition, environmental factors, and microbial imbalance [4].

Immunologically, AD is driven by a predominant type 2 helper (Th2) cell response, involving the release of cytokines like IL-4, IL-5, and IL-13 that promote IgE synthesis and eosinophilic inflammation. In chronic phases, Th1 and Th17 pathways may also contribute to persistent inflammation. Defects in filaggrin and other skin barrier proteins allow allergens and irritants to penetrate the skin, triggering immune activation [4,5].

Emerging evidence supports a “gut–skin axis,” where intestinal microbiota influences immune balance and cutaneous inflammation. Gut dysbiosis, characterized by reduced microbial diversity and lower abundance of species such as *Bifidobacterium*, has been linked to increased AD risk, particularly in infants [6]. Dysbiosis may reduce regulatory T cell (Treg) activity, which normally suppresses Th2-mediated allergic responses, and decrease production of short-chain fatty acids (SCFAs) that help maintain epithelial integrity. These changes lead to increased cytokine release and antigen sensitization [7].

AD symptoms are also often associated with anxiety, depression, and sleep disturbances, creating a psychosomatic cycle in which emotional distress further amplifies immune dysregulation. In children, this cycle can impact school performance, social relationships, and overall quality of life, underscoring the need to consider both physiological and psychosocial aspects of AD [8,9].

Probiotics are a promising intervention for pediatric AD, as they may restore microbial balance and promote immune tolerance by enhancing Treg activity, increasing IL-10 and TGF-β production, and reducing Th2-mediated responses. Microbial metabolites such as SCFAs can also strengthen epithelial barrier function and suppress inflammation, supporting the biological rationale for probiotic therapy [7]. While multiple randomized controlled trials have investigated probiotics in pediatric atopic dermatitis, results remain inconsistent regarding efficacy and strain specificity. AD severity is commonly assessed using the SCORAD scale (SCOring for Atopic Dermatitis), which combines the extent, intensity, and subjective symptoms of the disease. Some studies suggest that probiotics may reduce SCORAD scores in children, while other studies have found no significant difference between experimental and placebo groups [10,11].

This systematic review and meta-analysis of randomized controlled trials (RCTs) aims to compare the efficacy of various probiotic treatments (Lactobacilli, Bifidobacterium, etc.) as an intervention for children with AD. This review seeks to identify whether probiotic supplementation can decrease SCORAD indices in children and help alleviate symptoms.

## 2. Methods

### 2.1. Study Selection and Search Strategy

**Figure 1:**
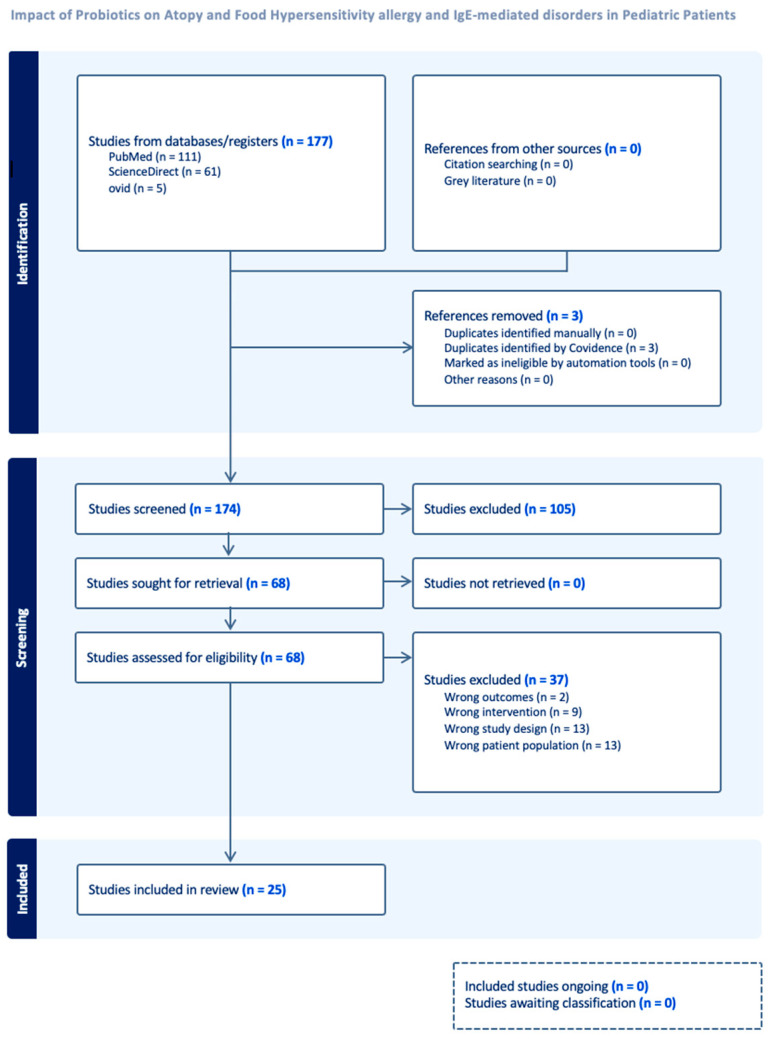
Study selection flow sheet—PRISMA flowchart of data extraction process.

This review met the guidelines for Preferred Reporting Items for Systematic Reviews and Meta-Analyses (PRISMA) as shown in Figure 1. The systematic review protocol was not registered in a public database such as PROSPERO, Cochrane, or Open Science Framework. Our search strategy involved three electronic databases, PubMed, ScienceDirect, and Ovid, through 9 October 2024, and identified randomized control trials of pediatric patients with an established diagnosis of atopic dermatitis that reported standardized SCORAD (scoring of atopic dermatitis) indices before and after intervention with probiotics or placebo. The full PubMed search strategy was as follows:

(“atopic dermatitis” [MeSH Terms] OR eczema [Title/Abstract] OR hypersensitivity [Title/Abstract] OR allergy [Title/Abstract])

AND (“probiotics” [MeSH Terms] OR probiotic*[Title/Abstract] OR “Lactobacillus” [Title/Abstract] OR “Bifidobacterium” [Title/Abstract])

AND (child*[Title/Abstract] OR pediatric [Title/Abstract] OR infant*[Title/Abstract] OR adolescent*[Title/Abstract])

AND (randomized controlled trial[pt] OR randomized [Title/Abstract] OR placebo [Title/Abstract])

NOT (animals [MeSH Terms] NOT humans [MeSH Terms]).

Equivalent search terms were adapted for ScienceDirect and OVID to account for differences in database indexing (e.g., Emtree terms).

The search included only English-language articles and did not restrict the country in which the study was conducted or the time frame of the study. Study selection was performed using Covidence software (www.covidence.org, accessed on 15 September 2024). Screening was performed by four reviewers against the Population, Intervention, Comparison, Outcomes, and Study (PICOS) scope for applicability to our research question found in Supplementary Materials File S1 accessed on 10 October 2024. The final screening included 174 articles, and 25 were included. Each article was reviewed independently by two authors screening at the title and abstract stage using the inclusion and exclusion criteria. All articles that were included were screened once more by two independent reviewers for specified criteria in the full text review stage, and conflicts were resolved through group consensus and discussion. Then, data extraction and risk of bias assessment were completed by two independent reviewers, and both authors discussed discrepancies in data extraction using Covidence.

### 2.2. Inclusion and Exclusion Criteria

After initial screenings, the Inclusion criteria were (1) pediatric patients, (2) patients with a formal diagnosis of atopic dermatitis, (3) patients treated with a probiotic regimen, and (4) randomized control trials. Exclusion criteria were (1) adult patients, (2) patients without an atopic dermatitis diagnosis, (3) observational and non-comparative studies, (4) articles not published in the English language, and (5) articles not available in full-length, peer-reviewed format.

### 2.3. Data Extraction

Data extraction was performed independently on each of the 25 studies. Study variables that were extracted included aspects such as sample size, recruitment period, how “probiotic” is defined, and the type of probiotic used, but were not limited to these variables. The full abstraction sheet can be found in Supplementary Materials File S2. During the preparation of this manuscript, the authors used ChatGPT (OpenAI, version GPT-4, released March 2023) for the purpose of learning how to interpret graphical data in instances where papers reported graphical changes in SCORAD scores. The authors have reviewed and edited the output and take full responsibility for the content of this publication.

### 2.4. Statistical Analysis

IBM’s SPSS Software version 30.0.0.0 was used to conduct forest plots using Hedge’s G test using mean difference values in experimental and mean difference in control groups, and calculated values such as effect size, I^2^ to assess heterogeneity, and the overall significance between experimental and control groups. The Hedge’s G test was utilized primarily to standardize measurement in standard deviation units due to varying scales and methods of measurements used in each study, such as mean SCORAD values. In situations where data were not complete, such as a lack of standard deviation in Weston 2005 or only medians being reported as in Isolauri 2000, they were excluded from our quantitative analysis [12,13]. Additionally, the J corrective factor in Hedge’s G adjusted for small sample sizes, which in the studies pooled varied from as few as 33 participants to 203 participants.

### 2.5. Risk of Bias Assessment

Study risk of bias assessment was evaluated for each study using the Quality Assessment published by Covidence, which is based on the Cochrane Risk of Bias (RoB 2) tool. This assessment was completed by each reviewer independently during the data extraction stage. This tool assesses six separate domains of bias, including sequence generation, allocation concealment, blinding of participants and personnel for all outcomes, blinding of outcome assessors for all outcomes, incomplete data for all outcomes, selective outcome reporting, and other miscellaneous sources of bias. This allowed for the exclusion of studies with a high risk of bias and served as a sensitivity analysis. The full quality assessment and risk of bias can be found in Supplementary Materials File S3.

## 3. Results

### 3.1. Study and Patient Characteristics

The systematic literature review identified 174 unique articles, with 25 full-text articles that were reviewed in detail. As outlined in Table 1, baseline characteristics of each study indicated varying usage of probiotics as intervention types, patient samples of varying sizes, and variability in study period and follow-up. Out of the 25 full text articles extracted, Lactobacilli strains were used in 23 studies, with a variety of subtypes including acidophilus, paracasei, rhamnosus, fermentum, pentosus, and johnsonii as notable strains used. Two studies used Bifidobacterium as an intervention, including Bifidobacterium lactis Bb-12 and a Bifidobacterium mixture including B longum, infantis, animalis, and breve subtypes. Each study corresponded to a study number, which was further referenced in the subsequent figures simply by number.

### 3.2. Effect of Probiotics on the Extent and Severity of AD

**Figure 2:**
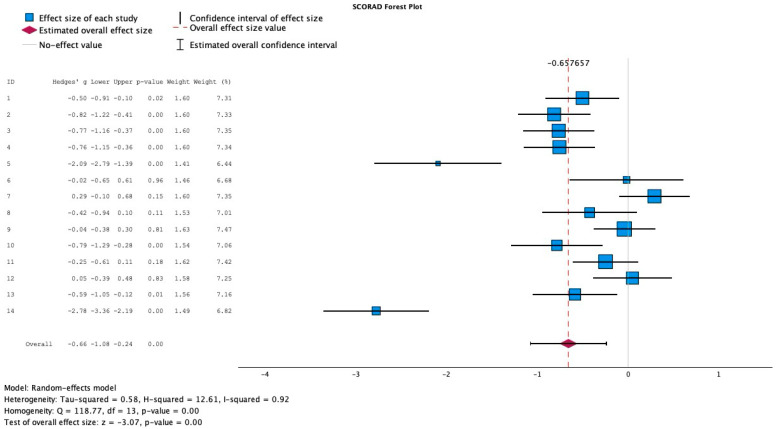
Effect size of SCORAD changes.

Out of the 25 full-text articles extracted, 14 articles reported SCORAD scores in pediatric atopic dermatitis patients. Hedge’s G forest plot generation, Figure 2, was used to capture effect sizes of each study and included pre-treatment control, pre-treatment experimental, post-treatment control, and post-treatment experimental mean SCORAD scores, as well as standard deviation scores of both post-treatment control and post-treatment experimental groups. The sample sizes of control groups and experimental groups were also captured. Two studies were excluded from forest plot generation given the inconsistencies in reporting mean SCORAD values scores. Isolauri 2000 was excluded from analysis as SCORAD scores were reported as median scores only, no mean values were reported [13]. Weston 2005 was excluded due to no standard deviation scores reported [12]. The Hedge’s G test was run across 12 studies reporting changes in SCORAD indices to calculate effect size and the value found was −0.65, which indicates a moderate to large negative effect size, with a negative sign indicating that when probiotics were added, there would be a correlated decrease in SCORAD score. Between-study heterogeneity was high (I^2^ = 92%), indicating considerable variability among included studies. Therefore, a random-effects model was used to account for inter-study variance.

### 3.3. Effect of Probiotics on IgE Levels

**Figure 3:**
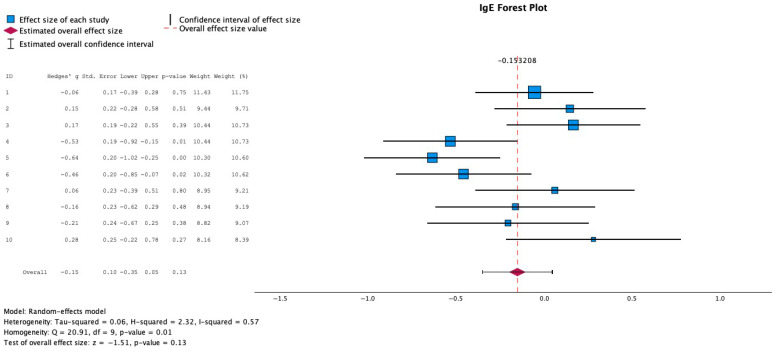
Effect size of IgE level changes.

Regarding Figure 3, a Hedge’s G test was run to look at the correlation between probiotics and IgE levels, and 6 studies were identified reporting IgE levels before and after the probiotic intervention, yielding an effect size of −0.15, meaning that there was a small effect size correlating a decrease in IgE levels with probiotic intervention. Thus, we can conclude that the relationship between probiotic intervention and a decrease in IgE levels is one of minimal impact, given the low absolute effect size value. When reporting IgE levels, all values were converted, assuming a normal logarithmic distribution, to the mean value of logs. This conversion into the mean value of logs was performed for each value to better handle any real-world data skew that may have existed in the IgE measurements. Log transformation also compressed large values, making the data more symmetric and closer to a normal distribution. A normal log distribution was assumed as the data was strictly positive, and the data grew exponentially.

### 3.4. Probiotic Strain Types: Phylogenetic Tree Representation

**Figure 4:**
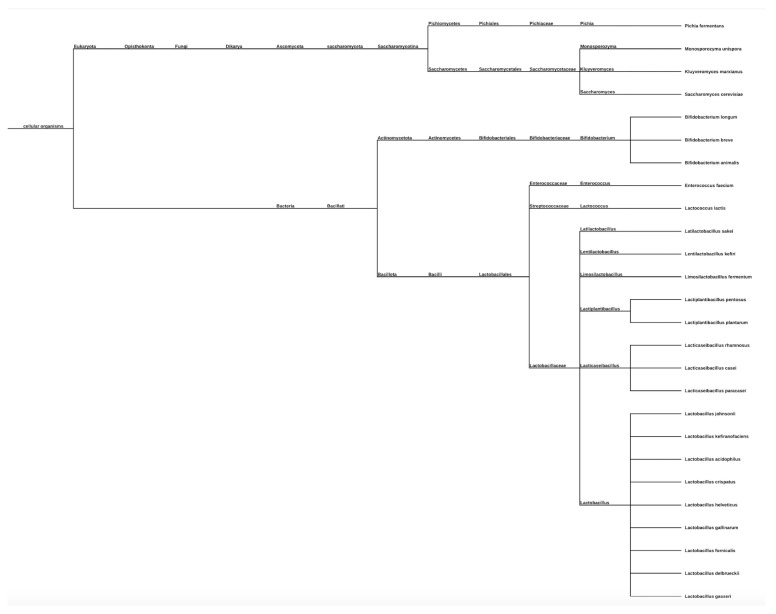
Phylogenetic tree of probiotic strain interventions w/subspecies.

As shown in Figure 4, there were a variety of different probiotic strains used as intervention types in each of the studies found in the broad literature search, and each species was categorized using the NCBI taxonomy generator, which recognized each species name and strain type and formulated a phylogenetic tree by comparing each strain to one another.

### 3.5. Mean Difference in SCORAD Change Across Studies

**Figure 5:**
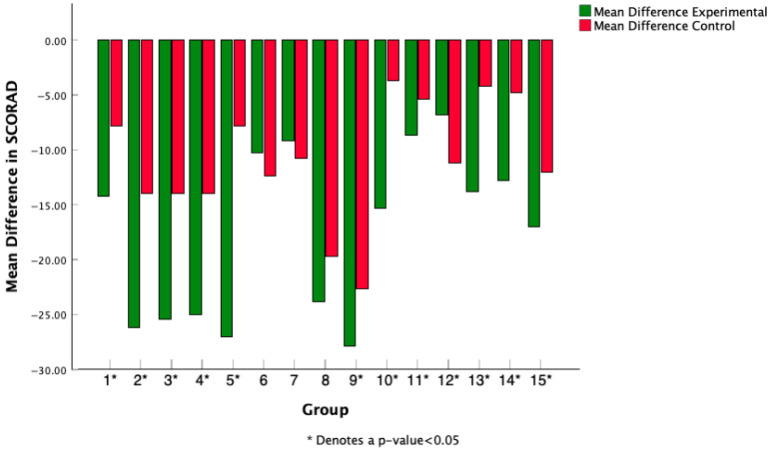
Mean difference in SCORAD across treatment and control groups.

Each study from Table 1 was mapped to a numerical value, and the mean difference in SCORAD score was calculated pre- and post-probiotic intervention for all 15 studies reporting SCORAD scores. Weston 2005 was excluded as standard deviation values post-treatment were not provided [12]. Mean differences were graphed in Figure 5, and mean differences in control groups pre- and post-intervention were compared to treatment groups pre- and post-intervention. These were mean differences captured within groups (control and treatment) to show how SCORAD indices changed over the course of treatment. Out of 15 studies identified reporting SCORAD and mean SCORAD difference values, 12 studies reported statistically significant differences between control and experimental groups over the course of the probiotic intervention.

### 3.6. Mean Difference in IgE Change Across Studies

**Figure 6:**
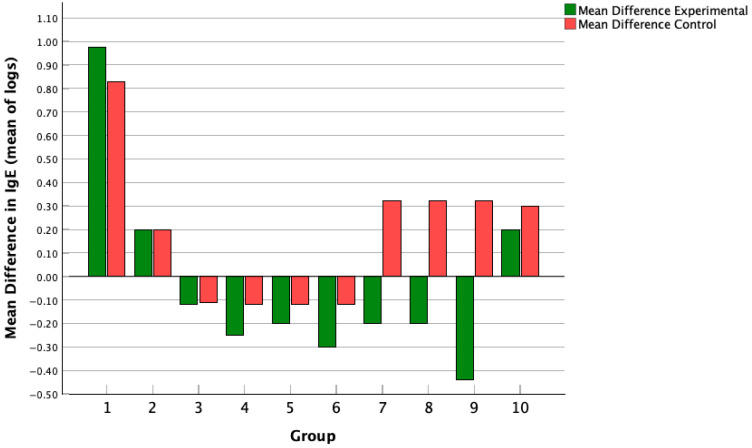
Mean difference in IgE across treatment and control groups.

Figure 6 shows the mean difference in IgE levels in control groups pre- and post-intervention compared to treatment groups pre- and post-intervention, and across 6 studies with 10 different subgroups. There were no study groups that showed a statistically significant difference between intervention and placebo.

### 3.7. Single vs. Combined Probiotic Strain vs. Intervention vs. Placebo SCORAD Scoring

The Fisher Exact Test was used to test for association between using a single or multiple strains and statistically significant differences in SCORAD levels after intervention. The *p*-value was 0.505. This test was used to assess the nominal data. Furthermore, the Pearson Chi-Square was not used as the test failed to meet the minimum expected count values. The 2 × 2 table indicated 9 statistically significant studies and 3 non-significant studies with a single strain used, and 1 statistically significant study and 1 non-significant study with combined strains used.

A random-effects model and Hedge’s G forest plot calculated the effect size of each study, capturing the change in SCORAD score with probiotic intervention, yielding an effect size of −0.65, indicating a moderate-to-large clinical impact of decreased SCORAD with probiotic intervention.

A random-effects model and Hedge’s G forest plot calculated the effect size of each study, capturing the change in IgE score with probiotic intervention, yielding an effect size of −0.15, indicating a small clinical impact of probiotics, causing a decrease in IgE.

The phylogenetic tree outlines each probiotic strain intervention included in data collection and subsequent analysis, with each strain organized by subspecies type. 

Mean difference in SCORAD treatment score was calculated pre- and post-treatment for both the experimental and control groups and reported across each study, with * for studies that found a statistically significant difference between experimental and control groups.

The mean difference in IgE was calculated pre- and post-treatment for both the experimental and control groups and reported across each study, with * for studies that found a statistically significant difference between experimental and control groups.

### 3.8. Risk of Bias Assessment of Results

The risk of bias was assessed using the Cochrane Risk of Bias (RoB 2) tool. Each of the 25 full-text studies was evaluated independently by the reviewers, and all papers were found to have a low risk of bias. This was primarily because each study was a randomized, double-blind controlled trial, with both probiotic and placebo treatments matched for characteristics such as appearance, taste, smell, and packaging, and further randomized to participant groups using computer-generated approaches. Additionally, if participants underwent protocol violation or discontinued treatment at any juncture, each study reported this, and their results were not included in the final data and data analysis.

### 3.9. Assessment of Publication Bias

**Figure 7:**
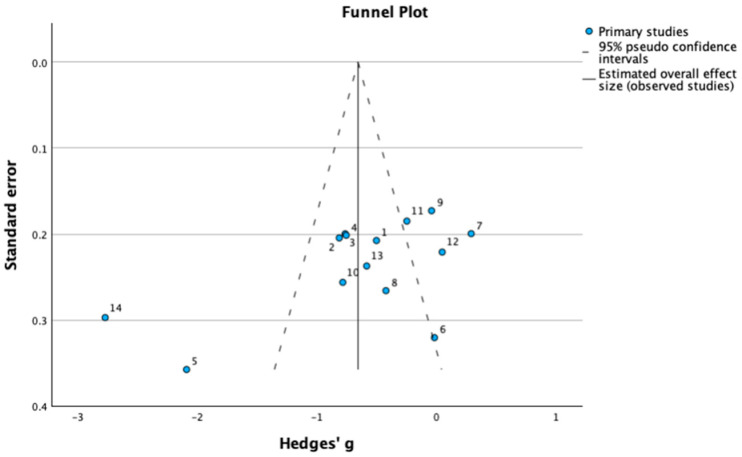
Funnel Plot for SCORAD Hedges’ g.

A funnel plot was created to systematically assess publication bias, shown in Figure 7. Egger’s regression test, presented in Table 2, showed a non-significant intercept with a (β = 1.38, *p* = 0.13), which indicates no evidence of funnel plot asymmetry and thereby no substantial evidence of publication bias. Although the slope coefficient was significant (*p* = 0.03), this suggests that study size was associated with effect magnitude more reflective of heterogeneity than true publication bias.

### 3.10. Sensitivity Analysis

The results of the leave-one-out analysis (Table 3) indicate that no single study had a disproportionate impact on the pooled effect size. The overall Hedge’s g ranged from −0.49 to −0.73 when each study was removed, remaining consistent in direction and magnitude, and confirming the robustness of our findings.

## 4. Discussion

Environmental factors have long been debated in the field of allergy and immunology as a potential therapeutic intervention for pediatric patients, particularly with the rise in C-sections, antibiotic use, a diet full of saturated fats and low fiber, and reduced omega-3 fatty acids and vitamin D, all contributing to gut dysbiosis [11]. Gut dysbiosis and changes to microbiota have been shown to play a role in allergic diseases such as eczema, asthma, and food allergies. Altered fecal microbiomes were associated with food allergies in infants, with specific food allergy subtypes associated with increased incidence in infants, and reduced bacterial diversity at the 1-month and 12-month mark was inversely associated with allergic sensitization, as evidenced by increased peripheral eosinophils and allergic rhinitis symptoms [36,37]. Furthermore, associations between altered microbiota profiles and atopic dermatitis have been evidenced with a study by Abrahamsson and colleagues, who looked at microbial diversity using 16S rDNA sequencing on stool samples of infants at 1 week, 1 month, and 12 months of age, and found decreased diversity of total microbiota during the first month of life and increased association with atopic eczema [38].

The relationship between microbiome shifts and subsequent allergic sequelae is evident, and probiotics may be a promising therapeutic additive on the horizon, either as an adjunct therapy or for a curative effect. Our literature search identified 174 articles that were screened using search criteria, and a total of 25 articles were extracted, focusing on pediatric patients with a pre-established diagnosis of atopic dermatitis and differing probiotic interventions that were delivered to patients, either in powder, capsule form, or added into formula in comparison to formulations without probiotics. Probiotics represent the optimal intervention target compared to other microbiome-modulating approaches such as prebiotics, synbiotics, or dietary modifications, as probiotics interact with both the gut and skin to induce tolerogenic immune responses, which is particularly relevant in atopic dermatitis, which is characterized by Th2-mediated inflammation; whereas, prebiotics rely on existing microbiota to produce beneficial effects. Additionally, looking into current data, probiotics have the largest number of randomized controlled trials looking into atopic dermatitis with consistent evidence of reproducible reduction in SCORAD scoring; whereas prebiotics and other synbiotic methods have not been as consistently reproducible, leading to less predictable clinical outcomes.

Probiotics are widely available over-the-counter in multiple formulations such as powders, capsules, or incorporated into infant formulas [39]. Patient perception is generally favorable, with studies reporting that many users perceive benefits [40]. Although there is insufficient evidence to determine the cost-effectiveness of probiotics for treating AD, probiotics can be considered a non-pharmaceutical option to complement conventional treatments.

Regarding meta-analysis, 14 articles were identified looking at changes in SCORAD score, with 12 studies reporting mean and standard deviation SCORAD scores pre- and post-intervention, and a Hedge’s G forest plot was performed to estimate the effect size of each study, yielding a moderate to large clinical impact of −0.65. Six studies were identified looking at changes in IgE levels pre- and post-probiotic intervention, and a Hedge’s g forest plot was also created, yielding an effect size of −0.15. A Hedge’s G test was used because this test captures the standardized mean difference and adjusts for small sample bias, which was the case across many studies gathered. The Fisher Exact Test was performed to test for an association between using a single or multiple strains and statistically significant differences in SCORAD levels after intervention. The *p*-value was 0.505, suggesting no significant correlation between the number of strains used and statistically significant SCORAD score changes pre- and post-intervention. This test was used to assess the nominal data. Furthermore, the Pearson Chi-Square was not used as the test failed to meet the minimum expected count values.

The pooled analysis demonstrated substantial heterogeneity (I^2^ = 92%), reflecting differences across trials in probiotic strains, intervention duration, and baseline disease severity. While it is true that high heterogeneity warrants caution when interpreting the pooled estimate, the consistent direction of effect toward improvement in SCORAD scores underscores a potential legitimate benefit. To address and mitigate this heterogeneity, future studies with standardized probiotic formulations and outcome measures should be utilized for subsequent meta-analyses.

The forest plot reporting changes in IgE levels could potentially not report the most accurate shift in IgE based on probiotic intervention, as it has been reported that IgE levels, in addition to cytokine levels, are long-term measures of well-being and often do not change drastically in a short few months’ intervention, as these studies reported.

Additionally, while the meta-analysis demonstrated reductions in SCORAD scores, the pooled standardized mean difference (Hedges’ g = −0.65) represents a moderate statistical effect, which may not correspond to a clearly defined clinically meaningful threshold for symptom improvement. The substantial heterogeneity observed across probiotic strains, treatment durations, and pediatric populations further limits generalizability. Hence, these findings should be interpreted as indicative rather than definite, and future long-term studies that keep an emphasis on standardized probiotic formulations and outcome measures are needed to determine whether short-term improvements translate into long-term clinical benefit in the pediatric population.

While other reviews have addressed probiotics in atopic dermatitis, they differ from our study in both scope and methodology. Wang et al. (2023) focused on prevention rather than treatment; Fijan et al. (2023) analyzed only Lactobacillus strains; and Xue et al. (2023) included 10 pediatric RCTs published up to 2022 [41,42,43]. Our review incorporates 12 pediatric RCTs published through October 2024, analyzes both single and multi-strain probiotic interventions, and additionally evaluates immunologic biomarkers (IgE, cytokines) alongside clinical outcomes (SCORAD). These updates make our review both broader in evidence coverage and more current, providing an essential pediatric-treatment-focused synthesis for clinicians and researchers.

### Limitations

A major limitation in methods to note would be the use of Hedge’s G as a data-capturing method. While methodologically valid in the case of small sample sizes given across studies, the mean difference in SCORAD indices may have been utilized to provide a direct assessment of the magnitude of improvement in SCORAD score. This contributed to the considerable heterogeneity that was present (I^2^ = 92%), limiting the precision of pooled estimates and precluding subgroup or meta-regression analyses due to the small number of comparable studies. Another limitation found while creating forest plots to capture the overall effect size was the differing study periods of each study included in the analysis. Regarding SCORAD effect size, many studies had study periods of 12 weeks, with a few others at 8 weeks and 16 weeks, which could have impacted how the effect size was calculated using a random effects model, as the study period may have been a confounding variable impacting SCORAD score results. Study follow-up periods ranged from 4 to 24 weeks with various follow-up schedules in place, also making this temporal dimension clinically relevant to the effect of probiotic intervention; however, this was not controlled for in statistical analysis, and this limitation must also be considered.

Additionally, there was a limited sample of studies, with twelve studies used to calculate SCORAD effect size, and six studies used to calculate IgE effect size. This could hinder further interpretation of results, as with a limited sample size of studies, conclusions may not be the most inclusive of all studies. Regarding the mean differences in IgE levels pre- and post-control and intervention, there were no statistically significant differences found between groups in each of the ten study groups identified across six different studies. This could be due to the fact that IgE levels often are a long-term diagnostic measure of allergy and may not shift as rapidly in the short-term interventional exposure time as seen across each study [44]. Study periods ranged from 12 to 36 weeks, furthermore suggesting the shorter-term course of the intervention administered.

Further data analysis could have been performed, examining probiotic type and conducting subgroup analyses of the *p*-values specific to each probiotic strain. However, of the 25 studies identified, 23 studies investigated Lactobacilli strains, and only 2 studies examined Bifidobacterium, potentially skewing the conclusions that could be made about Lactobacillus vs. Bifidobacterium efficacy, given a much larger sample size for Lactobacilli bacterial intervention.

## 5. Conclusions and Future Directions

Probiotics’ popularity within research and amongst general consumers has led to a variety of varying opinions on their true impacts. However, as it pertains to atopic dermatitis, this systematic review and meta-analysis suggests that probiotic supplementation may be associated with modest improvements in atopic dermatitis severity among pediatric patients, as reflected by reductions in SCORAD scores. However, these findings should be viewed as preliminary evidence given the limited number of available trials, variability in probiotic strains, and differences in study design and duration. Therefore, probiotics should remain as a potential adjunctive option, warranting further validation through large, well-controlled, strain-specific randomized trials before routine clinical implementation can be recommended.

## Figures and Tables

**Table 1:** Baseline characteristics of all studies.

Author, Year	Study #	Probiotic Used	Sample Size (*n*)	Measured SCORAD (Yes/No)	SCORAD Mean (SD) Pre; Post Intervention	End of Study Period	Follow-Ups (Weeks)
Gerasimov 2010 [14]	1	*L. acidophilus* DDS-1, *B. lactis* UABLA-12 with fructo-oligosaccharide	96	YES	42.1 (12.6); 27.9 (10.5)	Week 8	Week 2, 4, 8
Wang 2015 [15]	2, 3, 4	LP—*Lactobacillus paracasei* GMNL-133 (LP), 2 × 10^9^ colony-forming units (cfu) qd.; LF—*Lactobacillus fermentum* GM090 (LF), 2 × 10^9^ colony forming units (cfu) qd.	104	YES	LP: 50.93 (19.42); 25.48 (21.73) LF: 52.25 (16.85); 27.23 (18.1) LP + LF: 51.9 (18.9); 25.76 (19.23)	Week 12	Week 16
Navarro-López 2018 [16]	5	*Lactobacillus johnsonii* EM1 (Lj EM1)	50	YES	33.8; 6.8	Week 12	Week 4
Nermes 2011 [17]	6	*Lactobacillus rhamnosus* GG (LGG)	39	YES	27.9; 17.6	Week 12	None reported
Grüber 2007 [18]	7	*Lactobacillus rhamnosus* GG (LGG)	102	YES	34.5 (4.76); 25.35(15.13)	Week 12	Week 4, 8, 12
D’Auria 2021 [19]	8	*Lactobacillus paracasei* CBA L74	58	YES	42.5; 18.7	Week 12	Week 4, 8, 12
Cukrowska 2021 [20]	9	50% of *Lactobacillus casei* ŁOCK 0919, 25% of *Lactobacillus rhamnosus* ŁOCK 0908, 25% of *Lactobacillus rhamnosus* ŁOCK 0900 (Latopic^®^, Biomed S.A., Cracow, Poland).	134	YES	40.4 (20); 17.6 (14.8)	Week 12	Week 36
Jeong 2020 [21]	10	*Lactobacillus rhamnosus* (IDCC 3201, isolated from the feces of a Korean breast-fed infant, repeated heat-treated and incubated, RHT3201	66	YES	38.15; 24.25	Week 12	Week 6
Han 2012 [22]	11	*Lactobacillus plantarum* CJLP133	118	YES	30.6 (7.7);	Week 16	Week 14
					20.4 (12.6)		
Ahn 2020 [23]	12	*Lactobacillus pentosus*	82	YES	30.4(8.6);	Week 12	None reported
					23.6 (11)		
Woo 2010 [24]	13	*Lactobacillus sakei* KCTC 10755BP supplementation	75	YES	42.6; 28.8	Week 12	Week 6
Carucci 2022 [25]	14	*Lacticaseibacillus rhamnosus* GG	100	YES	Group A	Week 16	Week 16
					29.8 (12.1);25.0 (2.5)		
					Group B		
					30.8 (12.8);18.0 (2.5)		
* Weston 2005 [12]	15	*Lactobacillus fermentum* VRI-033 PCC	56	YES	40.8; 23.8	Week 8	Week 2, 4, 8, 16
** Isolauri 2000 [13]	16	*Bifdobacterium lactis* Bb-12 or Lactobacillus strain GG (ATCC 53103)	18	YES	Not reported.	Week 8	None reported
Huang 2018 [26]	17	*Lactobacillus paracasei* (LP), *Lactobacillus fermentum* (LF), both (LP + LF)	147	NO	Not reported.	Week 12	Week 4, 8, 12, 16
de Araujo 2017 [27]	18	*Lb. helveticus*, *Lb. kefiranofaciens*, *Lb. para-casei*, *Lb. kefiri*, *Lb. delbruecki*, *Lb. acidophilus*. *Lb. crispatus, Lb. gallinarum*, *Lb. fornicalis*, *Bifidobacterium breve*, *Lactococcus lactis*, *Enterococcus faecium*, *Kluyveromyces marxianus*, *Kazachstania unispora*, *Pichia fermentans* and *Sach. cerevisiae*.	60	NO	Not reported.	Week 16	Week 8
Basturk 2020 [28]	19	*Lactobacillus rhamnosus* GG (LGG)	106	NO	Not reported.	Week 4	None reported
Chen 2010 [29]	20	*Lactobacillus gasseri* A5	105	NO	Not reported.	Week 8	None reported
Torii 2011 [30]	21	*Lactobacillus acidophilus*	50	NO	Not reported.	Week 8	Week 4
Jerzyńska 2016 [31]	22	*Lactobacillus rhamnosus* GG (LGG)	50	NO	Not reported.	Week 20	None reported
Hol 2008 [32]	23	*Lactobacillus casei* CRL431 and *Bifidobacterium lactis* Bb-12	119	NO	Not reported.	Week 24	Week 48
Miraglia Del Giudice 2017 [33]	24	*Bifidobacterium* mixture (*B. longum* BB536, *B. infantis* M-63, *B. breve* M-16V)	40	NO	Not reported.	Week 8	None reported
Wang 2004 [34]	25	*Lactobacillus paracasei*-33 (LP-33)	80	NO	Not reported.	Week 4	None reported
Brouwer 2006 [11]	26	*Lactobacillus rhamnosis* (NP-Lrh) or Lactobacillus GG (NP-LGG)	33	NO	Not reported.	Week 12	Week 4, 8, 12
Anania 2021 [35]	27	mixture of *Bifidobacterium animalis* subspecies of Lactis BB12 and *Enterococcus faecium* L.	203	NO	Not reported.	Week 12	None reported

* Weston 2005 was excluded due to no standard deviation scores reported [12]. ** Isolauri 2000 was excluded from analysis as SCORAD values were reported as median scores only [13].

**Table 2:** Egger’s Regression Table for SCORAD.

Egger’s Regression-Based Test ^a^						
Parameter	Coefficient	Std. Error	t	Sig. (2-Tailed)	95% Confidence Interval	
					Lower	Upper
(Intercept)	1.378	0.8404	1.640	0.127	−0.453	3.209
SE ^b^	−8.651	3.5014	−2.471	0.029	−16.280	−1.022

^a^. Random-effects meta-regression. ^b^. Standard error of effect size.

**Table 3:** Leave-One-Out Sensitivity Table for SCORAD.

Leave-One-Out Sensitivity Analysis														
Excluding Study	1	2	3	4	5	6	7	8	9	10	11	12	13	14
Hedge’s g	−0.67	−0.65	−0.65	−0.65	−0.56	−0.70	−0.73	−0.68	−0.71	−0.65	−0.69	−0.71	−0.66	−0.49
*p*-value	0.00	0.01	0.01	0.01	0.01	0.00	0.00	0.00	0.00	0.01	0.00	0.00	0.00	0.00

## Data Availability

The data that support the findings of this study are publicly available and can be accessed online without any restrictions.

## Supplementary Materials

The following supporting information can be downloaded at: https://www.mdpi.com/article/10.3390/medicina61122090/s1, File S1. PICOTS Eligibility Criteria. File S2. Data Extraction Tables. File S3. Risk of Bias Tables.
