# Supplementary material for: Impact of Probiotics on Atopic Dermatitis in Pediatric Patients: A Systematic Review and Meta-Analysis
Source: Medicina (Kaunas). 2025 Nov 24;61(12):2090. doi: 10.3390/medicina61122090 (PMC12735213; doi:10.3390/medicina61122090)
Supplement: Supplementary file 1 [file medicina-61-02090-s001.zip › File S1.pdf]

File S1. PICOTS Eligibility Criteria

**Population**	- Inclusion:<br>- Pediatric patients (under 18) that were formally diagnosed with atopic dermatitis<br>- Patients that were subjected to a probiotic regimen protocol compared to standard of care<br>- Randomized control trials<br>- Exclusion:<br>- Patients who were not diagnosed with atopic dermatitis<br>- Adult patients<br>- Non-comparative studies/observational studies<br>- Studies not reporting atopy-related diagnosis<br>- Adult studies<br>- Studies not reported in the English language<br>- Articles not in full-length, peer-reviewed format
**Intervention**	- Inclusion:<br>- Any probiotic regimen intervention<br>- Exclusion:<br>- All other treatments that were not placebo or standard of care
**Comparator**	- No comparator needed or standard of care
**Outcomes**	- Efficacy outcomes analyzed:<br>- Scoring Atopic Dermatitis (SCORAD)
**Timing**	- Interventions with any follow-up period were included
**Setting**	- Interventions in any care setting were included
